# The impact of COVID-19 Vaccination in Iranian elderly: 7 percent of all-cause deaths reduced by vaccinating 2 percent of population; letter to editor 

**Published:** 2022

**Authors:** Sara Jambarsang, Moslem Taheri Soodejani

**Affiliations:** *Center for Healthcare Data Modeling, Departments of Biostatistics and Epidemiology, School of public health, Shahid Sadoughi University of Medical Sciences, Yazd, Iran*

**To the editor**

We read with interest the article recently published in Gastroenterology and Hepatology from Bed to Bench entitled “The impact of COVID-19 Vaccination in Iranian elderly: 7 percent of all-cause deaths reduced by vaccinating 2 percent of population” (1). The aim of the paper was to predict the impact of vaccination of old population on all-cause deaths based on time series model. 

We congratulate the authors for their valuable work, but there are fundamental criticisms of this conclusion.

First, at the beginning of the Covid-19 pandemic there was underreport of Covid-19 infection and death, according to studies. Among these studies, according to Ghaffari et al., Quoted by Iranian health officials, half of the suspected cases were not admitted to the hospital due to lack of test kits (2), however, these patients based on their clinical signs for COVID-19 are treated. Therefore, part of the deaths due to Covid-19 was recorded as other causes. With this explanation, the difference in deaths due to Covid 19 in the first year (before general vaccination) and the second (after general vaccination) is largely due to this underreport.

Second, death at the onset of the pandemic occurred faster due to a lack of knowledge about the disease. So comparing these two time periods can be misleading even in terms of deaths associated with Covid 19. It is probably best to compare deaths in proportion to the population as well as the standard population to the years before the pandemic. Therefore, the time period selected to fit the time series model is not a good representative for predicting and estimating the expected amount of difference. On the other hand, all the parameters of the model, including the time lag of the effects of an intervention on the outcome, must be quite clear (the effect of vaccination on deaths due to Covid 19 itself is somewhat time-consuming). If we want to compare, it is better to use the death rate in previous years as a basis for prediction, such as the study of Kelly et al., Which used 2015 as a basis (3).

Third, the study design was cross-sectional. Xu et al., in a cohort study considering both vaccinated and unvaccinated groups, stated that from December 2020 to July 2021, recipients of the Covid-19 vaccine were adjusted for age, sex, race, ethnicity, and non-Covid-19 mortality study. They had less than unvaccinated people. However, they do mention some major limitations to their cohort study. They believe that some observational effects are not possible in an observational study, so the effects of some disruptors, such as basic health status, underlying conditions, health care use, and socioeconomic status, have not been considered. Second, lower non-Covid-19 mortality rates in vaccinated groups indicate that Covid-19 vaccines are inherently healthier or have lower risk behaviors. Third, although Covid-19-related deaths were excluded, the causes of death were not assessed. It is possible that the algorithm used may have categorized some of the deaths associated with Covid-19 due to lack of testing or incorrect individual mortality studies (4).

Also, the reduction in deaths in this age group can be due to other causes of death such as reduced traffic and urban accidents, the impact of air pollution leading to the death of the elderly due to lockdown. However, Xu et al In their study suggested that differences in mortality due to other causes may have increased or decreased additional mortality from other diseases due to unprecedented public health measures implemented to control the epidemic. (4). On the other hand, a few months after the beginning of the pandemic, extensive activities in the field of self-care and other care for the elderly were considered, which was effective in reducing the incidence of Covid-19 in these people.

Finally, according to the available evidence, it is hasty to conclude about the effects of Covid-19 vaccination on deaths from all causes, although the effect of this measure on reducing Covid-19 deaths is clear.

## Conflict of interests

The authors declare that they have no conflict of interest.
